# Effect-Based Approach to Assess Nanostructured Cellulose Sponge Removal Efficacy of Zinc Ions from Seawater to Prevent Ecological Risks

**DOI:** 10.3390/nano10071283

**Published:** 2020-06-30

**Authors:** Giulia Liberatori, Giacomo Grassi, Patrizia Guidi, Margherita Bernardeschi, Andrea Fiorati, Vittoria Scarcelli, Massimo Genovese, Claudia Faleri, Giuseppe Protano, Giada Frenzilli, Carlo Punta, Ilaria Corsi

**Affiliations:** 1 Department of Physical, Earth and Environmental Sciences and INSTM Local Unit, University of Siena, 53100 Siena, Italy; giulia.liberatori@student.unisi.it (G.L.); grassi23@student.unisi.it (G.G.); giuseppe.protano@unisi.it (G.P.); 2 Department of Clinical and Experimental Medicine-Section of Applied Biology and Genetics, University of Pisa, 56126 Pisa, Italy; patrizia.guidi@unipi.it (P.G.); margherita.bernardeschi@unipi.it (M.B.); vittoria.scarcelli@unipi.it (V.S.); massimo.genovese@unipi.it (M.G.); 3 Department of Chemistry, Materials, and Chemical Engineering “G. Natta” and INSTM Local Unit, Politecnico di Milano, 20131 Milano, Italy; andrea.fiorati@polimi.it (A.F.); carlo.punta@polimi.it (C.P.); 4 Department of Life Sciences, University of Siena, 53100 Siena, Italy; faleric@unisi.it

**Keywords:** cellulose-based nanosponges, zinc, seawater, ecotoxicity, effect-based approach, remediation

## Abstract

To encourage the applicability of nano-adsorbent materials for heavy metal ion removal from seawater and limit any potential side effects for marine organisms, an ecotoxicological evaluation based on a biological effect-based approach is presented. ZnCl_2_ (10 mg L^−1^) contaminated artificial seawater (ASW) was treated with newly developed eco-friendly cellulose-based nanosponges (CNS) (1.25 g L^−1^ for 2 h), and the cellular and tissue responses of marine mussel *Mytilus galloprovincialis* were measured before and after CNS treatment. A control group (ASW only) and a negative control group (CNS in ASW) were also tested. Methods: A significant recovery of Zn-induced damages in circulating immune and gill cells and mantle edges was observed in mussels exposed after CNS treatment. Genetic and chromosomal damages reversed to control levels in mussels’ gill cells (DNA integrity level, nuclear abnormalities and apoptotic cells) and hemocytes (micronuclei), in which a recovery of lysosomal membrane stability (LMS) was also observed. Damage to syphons, loss of cilia by mantle edge epithelial cells and an increase in mucous cells in ZnCl_2_-exposed mussels were absent in specimens after CNS treatment, in which the mantle histology resembled that of the controls. No effects were observed in mussels exposed to CNS alone. As further proof of CNS’ ability to remove Zn(II) from ASW, a significant reduction of >90% of Zn levels in ASW after CNS treatment was observed (from 6.006 to 0.510 mg L^−1^). Ecotoxicological evaluation confirmed the ability of CNS to remove Zn from ASW by showing a full recovery of Zn-induced toxicological responses to the levels of mussels exposed to ASW only (controls). An effect-based approach was thus proven to be useful in order to further support the environmentally safe (ecosafety) application of CNS for heavy metal removal from seawater.

## 1. Introduction

The rapid industrialization and economic development of marine coastal areas have significantly increased the release of toxic pollutants, leading the marine environment to be considered a final sink [1]. Heavy metal pollution due to different sources and anthropogenic activities (sewages, ship and gas/oil platforms discharges, antifouling paints, landfill sites) still represents a critical issue in marine coastal waters, being responsible for ecological deterioration and for giving rise to health risks for humans and marine wildlife [2,3]. In order to achieve a Good Environmental Status following the Marine Strategy Framework Directive (MSFD, Directive 2008/56/EC) [4], heavy metals in European coastal waters should be kept below those concentrations that are able to cause toxicity to marine species (from mg L^−1^ down to µg L^−1^) (Environmental Quality Standard Directive 2013/39/EU) [5]. Zinc levels very often exceed regulatory limits in marine coastal areas, with concentrations of up to 10 ppm in both sediments and the water column. Although Zn(II) is known to pose a serious threat to humans and the environment, its worldwide production has increased in the last ten years, so that its presence in the aquatic environment is expected to grow [6]. Further sources of Zn(II) release into the sea include Zn-based antifouling paints and sunscreens (e.g., zinc oxide), as well as discharges from desalination plants and bilge water disposal [7,8]. In the ocean, dissolved Zn(II) undergoes a nutrient-like vertical profile and sediments represent a source to the water column. This will in turn significantly affect Zn(II)’s bioavailability, bioaccumulation and toxicity for marine species belonging to different trophic levels [9,10,11,12,13,14,15,16]. Zn(II) is also an essential element for selected marine species, therefore its levels in surface waters should not decrease below a certain concentration (µg L^−1^), which could limit phytoplankton growth [17].

The use of engineered nanomaterials (ENMs) was recently intended as a more attractive and convenient remediation solution compared to conventional ones, being less expensive and more effective, as well as environmentally and economically sustainable [18,19]. Their usage for cleaning up polluted soils and waters, termed “nanoremediation”, has made enormous progress over the last few decades [20]. However, the risks associated with their mobility and transformations once released into the environment are almost unknown [18]. To fill these scientific gaps whilst going back to a perspective of environmental and economic sustainability, research is moving towards the design of environmentally friendly ENMs which do not pose any risk for living beings and overall ecosystems [21,22,23]. In this direction, several contributions have been made to develop eco-friendly bio-based ENMs which have a high capacity to reduce environmental pollution and are suitable to be used onsite [24,25,26,27]. Among renewable sources, cellulose is receiving great attention, being an abundant biopolymer with good mechanical properties and numerous possibilities for chemical functionalization. It also represents a promising sustainable material, since its synthesis does not require the use of high temperatures and CO_2_ emissions are lower than those connected to the production of classical ENMs [22,23,28,29,30,31,32,33]. Moreover, due to the degree of branching that can be obtained, the area-to-volume ratio is very high, promoting a greater efficiency of adsorption, which can be increased by tailoring cellulose with suitable functional groups [34]. In 2015, for the first time, we reported a two-step thermal protocol for the synthesis of a novel family of cellulose-based ENMs which possessed superb performance in the adsorption of metals and organic contaminants [35]. It was obtained by the cross-linking of 2,2,6,6-tetramethyl-piperidine-1-oxyl (TEMPO)-oxidized and ultra-sonicated cellulose nanofibers (TOUS-CNFs) [36] in the presence of branched polyethyleneimine (bPEI), thus achieving a nanostructured cellulose sponge (CNS) suitable for heavy metal ion removal from water. CNS shows a 2D sheet-like morphology, characterized by a micro-porosity, which can be observed by the scanning electron microscopy (SEM) technique, and a nano-porosity, as revealed by an in-depth small angle neutron scattering (SANS) analysis [37]. More recently, the adsorption efficiency of CNS from seawater was revealed to be better than from freshwater, and their environmental safety (ecosafety) is achieved by exploiting an eco-design approach capable of combining life cycle assessment (LCA) and environmental risks [38,39,40]. To encourage the applicability of CNS as adsorbent materials for toxic heavy metal removal from seawater, here, we propose an ecotoxicological evaluation designed by an effect-based approach. Considering their proven ability to remove heavy metals from seawater [40], we aim to prove their efficacy in reducing Zn toxicity by measuring known Zn-induced toxicological responses, both at cellular and tissue levels, in a model marine species, namely the bivalve mollusk *Mytilus galloprovincialis*. Being a widely adopted marine model in ecotoxicology, *Mytilus sp.* has been historically used as a bioindicator in marine pollution monitoring (mussels watch programs) [41,42] due to its known ability to accumulate heavy metals in soft tissues, including Zn (ranging between µg-mg/kg wet weight), in proportion to their concentration in seawater [43,44,45,46]. The main pathway of Zn(II) uptake in *Mytilus* is through the gills, from which it is transported to the digestive system via the hemolymph [47,48,49,50]. Zn-induced biological responses/effects have been already well identified in marine mussels, both at cellular and tissue levels, and described in detail [51,52,53,54,55,56,57]. Being the first organs involved in Zn(II) uptake in bivalves, the gills and mantle are the main targets of Zn toxicity in mussels [58]. Zn damage to gills’ cilia impairs their movement but also affects their sensory and secretory activities, as well as shell growth and repair mechanisms [59]. Zn waterborne exposure (125 µg L^−1^) is able to induce histopathological alterations of mantle external epithelial cells and to increase mucous cells [60]. At the cellular level, lysosomal membrane destabilization has been documented in mussels’ hemocytes upon waterborne exposure to ZnCl_2_ (0.5 and 1 mg L^−1^, 7 days) [61]. DNA damage was reported to be either induced by reactive oxygen species (ROS) or by direct binding to DNA in mussels’ embryos which were exposed to Zn-contaminated sediments [62] and micronuclei and nuclear abnormalities in mussels’ gill cells as cyto-genotoxic biomarkers in pollution monitoring [63].

With the aim of using an effect-based approach to assess CNS’ ability to remove Zn(II) from seawater, lysosomal membrane stability (LMS) and DNA damage were investigated in mussels’ hemocytes and gill cells, as well as histopathological alterations on the mantle edge and external epithelial cells. Specimens of *M. galloprovincialis* were exposed to ZnCl_2_ contaminated artificial seawater (10 mg L^−1^ ASW) before and after CNS treatment, and two groups exposed to ASW (control) and CNS alone were also tested. Levels of Zn(II) were also measured in ASW in all experimental groups (ZnCl_2_, Zn-t CNS, CNS, ASW) at time zero and after 24 h of exposure.

## 2. Materials and Methods

### 2.1. CNS Synthesis, Characterization and Zn(II) Adsorption

TOUS-CNFs were prepared according to a standardized protocol reported by [38]. Briefly, 190 g of cotton linters cellulose, provided by Bartoli Spa paper mill (Capannori, Lucca), were dispersed in deionized water (2 L) and added to an aqueous solution (3.7 L) of 2,2,6,6-tetramethyl-piperidine-1-oxyl (TEMPO 2.15 g, 13.8 mmol) and KBr (15.42 g, 129 mmol) under vigorous stirring. While maintaining the stirring, NaClO (12.5% w/w aqueous solution, 437 mL) was slowly added to the mixtures and the pH value maintained in the range of 10.5–11 by using sodium hydroxide solution (4 M). The reaction was left stirring overnight and then acidified to pH 1–2 with concentrated HCl (37% w/w aqueous solution). The oxidized cellulose was collected by filtration on a sintered glass funnel and washed extensively with deionized water (5 × 2 L) and acetone (2 × 0.5 L, 99.9% purity). TOUS-CNFs were recovered by 84%. The carboxylic content of the oxidized cellulose was then colorimetric titrated (1.5 mmol/g of –COOH units). The obtained oxidized cellulose was dispersed in deionized water (3% w/v), a stoichiometric amount of NaOH was added and the mixture was sonicated at 0 °C for 30 min, using a Branson Sonifier 250 equipped with a 6.5 mm probe tip, working at 20 kHz in continuous mode, with an output power 50% the nominal value (200 W). An excess of HCl (aq, 1 M) was added in order to precipitate TOUS-CNFs that were collected by filtration on Büchner funnel with a sintered glass disc and further washed on the filter with deionized water (450 mL × 3 times).

**Figure 1:**
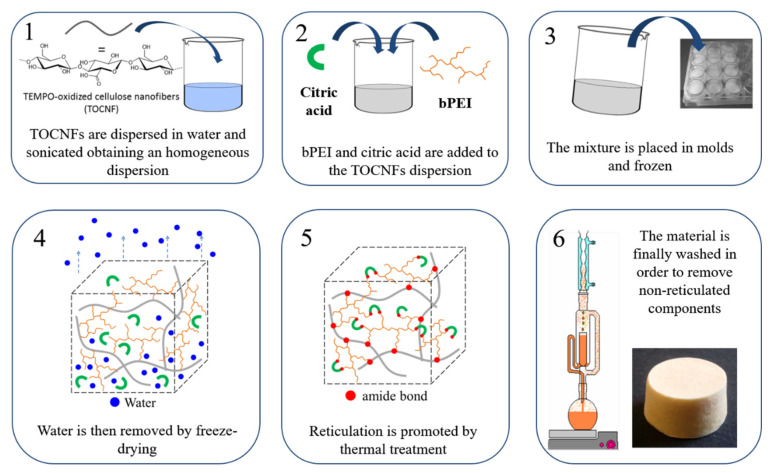
Synthetic procedure for the production of cellulose-based nanosponges CNS.

CNS were prepared according to the optimized procedure which was recently reported [40]. TOUS-CNFs were dispersed in an aqueous solution of 25 kDa bPEI (84 g of bPEI in 300 mL of water) and citric acid (23.8 g in 200 mL of water). The mixture was sonicated again for 10 min. The viscous gel obtained was transferred into a mold, frozen at −80 °C for 12 h and freeze-dried at −60 °C for 48 h. The cylindrical disks were thermally treated in an oven (103 °C, 12–16 h). The resulting xerogels were ground in a mortar and washed with water (6 × 150 mL, 1 h contact time for each cycle). The CNS synthetic procedure is summarized in Figure 1.

The particle sizes of the ground CNS powder were measured in the Laboratory Chemical Analysis (LAC) of Politecnico di Milano by means of a Malvern Mastersizer 3000 Particle Size Analyzer (Malvern, UK) with Fraunhofer modeling, which considers opaque non-spherical particles. The CNS powder was suspended under stirring in 500 mL of water to reach an obscuration level in the range of 8%–12%.

The adsorption ability of CNS towards Zn(II) from ASW was determined as follows. In a Falcon test tube, 12 mg (±0.2 mg) of CNS were dispersed in 15 mL of ZnCl_2_-contaminated solution at different metal ion concentrations (1.5, 15, 30, 45 and 90 ppm). In all cases, the mass of sorbent material per volume of solution was 0.8 mg mL^−1^. The test tubes were sealed and left at 25 °C and shacked for 24 h. Upon filtration (0.45 µm), the Zn(II) solutions were analyzed using Agilent Technologies Inductively Coupled Plasma Mass Spectrometry (ICP-MS, Agilent Technologies, Santa Clara, CA, USA) 7900, equipped with a MicroMist nebulizer and a spray chamber in a Peltier-cooled sample introduction system, in order to increase stability and consistency, followed by a shield torch system and temperature-controlled collision/reaction octopole ion guide cell, in order to provide interference removal in the helium collision mode. All analytical operations were carried out in compliance with method EPA 6020B 2014. Instrument calibration was carried out by standard solutions used as reference material, which were purchased from qualified suppliers (Exaxol Italia, Genova, Italy), and containing the analytes in a suitable concentration range.

The scanning electron microscopy (SEM) of the CNS was performed according to the method which has been described in detail [40]. Briefly, after Zn(II) adsorption, horizontal sections of CNS (half height) were fixed in aluminum stubs using graphite powder (Agar scientific, G3300 Leit C—conducting carbon cement) and placed in the oven at 50 °C (30 min) and coated with graphite (Emitech K450 apparatus, Quorum Technologies Ltd., Laughton, UK). SEM images were obtained using a Zeiss instrument Gemini Supra 40 model (accelerating voltage 20 kV, spot size 60 μm, Oberkochen, Germany) with an energy-dispersive electron probe X-ray (EDX) (Oxfod x-act).

### 2.2. Experimental Design of Effect-Based Study

#### 2.2.1. In Vivo Waterborne Exposure

Adult specimens of *M. galloprovincialis* (medium length of the valves 6.5 ± 0.5 cm) were purchased from an aquaculture farm (Sardinia, Italy) and shipped to the marine aquarium facility of the Department of Physical, Earth and Environmental Sciences of the University of Siena (Siena, Italy). Based on our previous experience, an acclimatization period of 48 h was sufficient for reaching a stable physiological condition for mussels [64]. Natural seawater (NSW) collected from a pristine area in Tuscany (Italy) was used for acclimatization and the following parameters were kept constant during the entire period: salinity 40% ± 1%, pH 8 ± 0.1, density 1.025 ± 0.001 g cm^−3^, conductivity 49 ± 1 mS cm^−1^ and temperature 18 ± 1 °C.

A set of two experiments was run: (i) a preliminary experiment was designed to select ZnCl_2_ sub-lethal effects concentration for mussels; (ii) a second experiment aimed to assess the CNS’ ability to reduce ZnCl_2_-induced toxicological responses/effects in exposed mussels. Both experiments were run in artificial seawater (ASW), prepared following [65], in order to test CNS’ performance in high ionic strength media and avoid inorganic and organic colloidal particles as well as other compounds naturally occurring in NSW.

In the preliminary experiment (i), mussels were exposed for 48 h to ZnCl_2_ at concentrations of 1, 10 and 100 mg L^−1^ in ASW, which were chosen based on the LC_50_ values (range 2–20 mg L^−1^) available in the literature for bivalve species [51,52,66,67,68,69,70]. A parallel group of mussels exposed to ASW only was used as a control. Mussels were placed in 6 L glass tanks in groups of 6 individuals per experimental condition, using a ratio of 1 mussel: 1 L ASW, and they were not fed during the experiment. The exposure medium was renewed every 24 h to maintain constant Zn(II) nominal exposure concentrations. At the end of the 48 h period, the mussels were removed from the tanks, the hemolymph was collected for lysosomal membrane stability (LMS) in hemocytes by a neutral red retention time assay (NRRT) (method described in detail in Section 2.2.2), and chromosomal damage was measured through the micronucleus test (MN) (method described in detail in Section 2.2.2).

In the second experiment (ii), the mussels were exposed for 48 h to ZnCl_2_ (10 mg L^−1^) in ASW (Zn), before and after CNS treatment (Zn-t CNS), and two groups, one exposed to ASW only (ASW) and one with only CNS (CNS), were prepared by following the same experimental conditions used for the Zn-contaminated ASW treatment. We applied a ratio of 1.25 g of CNS: 1 L of ASW for the CNS treatment, based on our previous findings on heavy metal adsorption from ASW [40]. Briefly, 1.25 g L^−1^ of CNS powder was incubated with ZnCl_2_-contaminated ASW (10 mg L^−1^) for 2 h at room temperature, under vigorous magnetic stirring in order to increase the contact between CNS and Zn (II) in the water medium. CNS alone were also incubated in ASW at the same ratio (1.25 g powder L^−1^ ASW) and further tested for reference. Both CNS treated mediums were then filtered at 0.45 µm using a cellulose filter to remove excess CNS powder, and the obtained solutions (Zn-t CNS and CNS alone) were tested with mussels. The experimental groups of ZnCl_2_ in ASW (10 mg L^−1^) and ASW only were also set up and tested. In summary, mussels (1 specimen: 1 L of ASW) were placed in 6L tanks (total of 6 individuals) and exposed to the following experimental solutions: ZnCl_2_, Zn-t CNS, CNS only and ASW for 48 h. Mussels were not fed during the experiment and all tested solutions were renewed every 24 h to maintain constant exposure conditions.

At the end of the 48 h period, the hemolymph was collected for the LMS NRRT assay (described in detail in Section 2.2.2) and chromosomal damage was measured through micronucleus (MN) frequency (described in detail in Section 2.2.2).

Gill arches and the full mantle were also removed with scissors, and single gill arches were processed for comet and cytome assays (described in detail in Section 2.2.2), while the mantle was processed for histological examination [71]. In addition, three independent samples of ASW (10 mL) from each tested solution (ZnCl_2_, Zn-t CNS, CNS only and ASW) were collected at time zero and after 24 h, and they were stored at 4 °C for analysis of the total Zn(II) levels, according to the method reported below (Section 2.3).

#### 2.2.2. Cellular Bioassays

For the NRRT assay [72], the hemolymph was withdrawn from the mussel’s adductor muscle using a sterile syringe (1 mL) pre-loaded with a buffer solution (20 mM 4-(2-hydroxyethyl)-1-piperazineethanesulfonic acid (HEPES), 436 mM NaCl, 53 mM MgSO_4_, 12 mM KCl, 10 mM CaCl_2_) to prevent hemocyte clotting. A 200 µL volume of hemocyte suspension was then placed in a coverslip (22 × 22 mm), incubated in the dark at 18 °C by adding 200 μL of neutral red dye solution (0.1 mg mL^−1^ NR in dimethyl sulfoxide (DMSO)). Excess NR was removed before observing slides under optical light microscopy (Olympus BX51, 80× magnification), and the percentage of cells showing loss of the NR dye from lysosomes was scored every 15 min. Three replicates of three different slides were analyzed, each one made by pooling the hemolymph from at least five individuals for each experimental group. Final scores were set at the time when 50% of hemocytes showed NR leaking from lysosomes and became round-shaped [58,73].

The MN frequency assay [74] was run on withdrawn hemolymph diluted in 10 mM ethylenediaminetetraacetic acid (EDTA) buffer saline solution. A 200 μL volume of hemolymph was placed in cold slides and incubated at −20 °C in methanol for 20 min first and in 6% Giemsa dye in MilliQ for another 20 min. MN frequency was scored every 1000 hemocytes under an optical light microscope (Olympus BX51, 80× magnification). Three replicates of three different slides were analyzed, each one made by pooling hemolymph from at least five individuals for each experimental group.

DNA primary damage, including DNA single and double strand breaks (SB) and alkali labile sites, was evaluated by the comet assay in mussels’ gill cells, according to the method previously described in [75]. Briefly, after dissection, gills were put in a tissue digestion solution (dispase/Hank’s Balance Salt Solution (HBSS)) at 37 °C for 20 min; then, the enzyme was inactivated. The resulting digestion product was filtered through a 100 μm mesh nylon filter and the cell suspension obtained was centrifuged at 2000 rpm for 5 min. Cell viability was evaluated by trypan blue exclusion and only cells displaying a cell viability >95% were included. The slide preparation and electrophoresis procedures were performed according to the protocol reported in [76]. The amount of DNA damage, expressed as the percentage of DNA migrated into the comet tail (% tail DNA), was assessed from at least 50 nuclei per slide (*n* = 2) per specimen, using an image analyzer (Kinetic Imaging Ltd., Komet, Version 5).

The pellets obtained from mussels’ gill cells were processed for the cytome assay according to [77]. One thousand cells with preserved cytoplasm per specimen were scored (500 per slide) in order to determine the frequency of MN, according to the criteria listed by Fenech [78]. Morphologically altered nuclei (i.e., incomplete MN, lobed or multiple nuclei, nuclei connected by chromatin bridges in the same cell) were scored on the same slides in parallel and were collectively reported. The frequency of total nuclear abnormalities (NA) (nuclear blebs (BL), included nuclear buds (NBUD), binucleated cells with nuclear bridges (NPB), notched nucleus (NT), circular nucleus (CIR), lobed nucleus (LB), and anisochromatic nuclei (AN) was observed (Figure S1). Apoptotic cells (APO) were also evaluated. For each experimental group, four specimens, with 2 slides per specimen and 500 cells per slide for a total of four thousand cells per experimental point, were scored.

#### 2.2.3. Mantle Histology and Histochemistry

Mantles were gently removed from the internal shells of mussels using scissors, and small sections of mantle edge were obtained with a scalpel (average 1 cm) and freshly observed under a Zeiss Stemi SV6 (8×–50× magnification) binocular stereo microscope at magnification (8×, 10× and 20×) by placing them in glass Petri dish.

The mantle edge sections for histological analysis were obtained by cutting the most external part of the mantle (average 1 mm) and fixing it in a modified Karnovsky solution (3% glutaraldehyde in 0.1 M sodium cacodylate buffer 0.9% sucrose, pH 7.2) for 2 h at room temperature. They were fully dehydrated in ethanol and embedded in resin (Kulzer Technovit 7100) and polymerized at 27 °C for 3 h. Serial sections (2 µm) were obtained with the LKB Ultratome Nova microtome and stained with haematoxylin and eosin (H&E) (pH 2.4, 10 min at 25 °C). As a contrast dye able to stain eosinophilic structures in various shades of red, pink and orange, 0.5% eosin Y in aqueous solution, acidified with acetic acid and filtered at 0.45 µm, was used for 1 min. Sections were then rinsed in distilled water for 10 min, air dried, sealed and analyzed under a Zeiss Axiophot epifluorescent microscope (80X) with AxioCamMRc5.

In order to detect mucosubstances, the specific dye, periodic acid Schiff–Alcian blue (PAS–AB), was used. The reaction between the periodic acid (PA) and the Schiff reagent stains the neutral mucopolysaccharides in purple, while the AB identifies the acid mucopolysaccharides in blue. Before being colored, the sections were placed for 20 min in 100% acetone in order to remove the resin. The sections were rinsed in tap water for 2 min and then placed in 1% PA in distilled water for 5 min. Sections were rinsed in tap water for 5 min and then the Schiff reagent was added for 1 h. After the Schiff reagent was removed, 0.5% Na_2_S_2_O_5_ in 1% HCl was placed for 10 min on the slides. Slides were rinsed in tap water for 5 min and 1% AB in 3% acetic acid were left for 30 min. Finally, the slides were rinsed in distilled water for 2 min, dried, sealed and analyzed under a Zeiss Axiophot epifluorescent microscope (80X) with AxioCamMRc5.

### 2.3. Quantification of Total Zn Levels in Tested Solutions 

The total amount of Zn was measured in each tested solution (ZnCl_2_, Zn-t CNS, CNS only and ASW), at time zero (*T*_0_) (soon after preparation) and after 24 h (*T*_24h_), as follows: 10 L of mussels’ exposure water was removed from each tank, placed in polyethylene plastic tubes and stored at 4 °C. Analysis was performed by ICP-MS using the Perkin Elmer NexION 350 spectrometer (Waltham, MA, USA). The analytical accuracy was checked through the determination of Zn(II) concentration in SLRS-6 (river water certified reference materials for trace metals and other constituents) and CASS-4 (nearshore seawater certified reference materials for trace metals and other constituents) of the National Research Council of Canada. Analytical precision was evaluated by means of the percentage relative standard deviation (% RSD) of five replicate analyses of each exposure water sample. Zn levels were expressed as mg L^−1^. Variations in pH in exposure waters were also recorded at time zero and after 24 h.

### 2.4. Statistical Analysis

The data that were obtained and represented as mean ± SD of at least 5 samples in triplicate were analyzed by GraphPad Prism software package 6. Statistical analysis for hemocyte tests was performed using one-way analysis of variance (ANOVA) plus the Bonferroni post-test. For the tests on gill cells, data from 4 specimens were analyzed by the multifactor analysis of variance (MANOVA) and the multiple range test (MRT) was performed in order to detect differences among experimental groups. For all data analyses, the statistical significance level was set at *p* < 0.05.

## 3. Results and Discussion

### 3.1. CNS Adsorption Performance of Zn(II) from ASW

**Figure 2:**
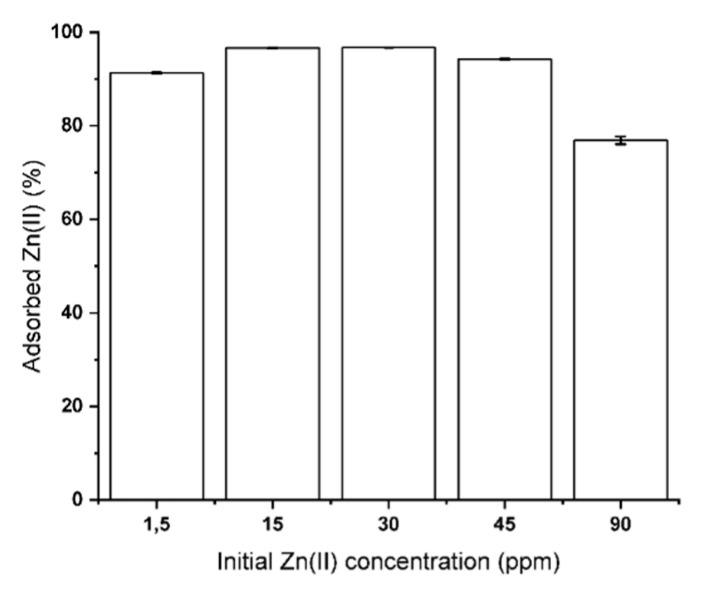
Cellulose-based nanosponges’ (CNS) adsorption efficiency of Zn(II) from mono-contaminated artificial seawater (ASW) at different ion concentrations. CNS amount: 0.8 g L^−^^1^.

The adsorption efficiency of Zn(II) from ASW was evaluated by contacting the mono-contaminated solution at different ion concentrations with ground CNS. The results reported in Figure 2 clearly show that, by operating at a CNS/ASW ratio of 0.8 g L^−1^, the abatement of Zn(II) ions in a range between 1.5 and 45 ppm was higher than 90%, with a maximum adsorption capability of about 100 mg per g of adsorbent material [40]. As described in our previous works [35,40], the adsorption efficiency can be ascribed to the chelating action of free amino groups present in the CNS network. As a consequence, the slight basic pH (8) of seawater allows the prevention of the protonation of amino groups, permitting them to completely exploit their chelating action.

**Figure 3:**
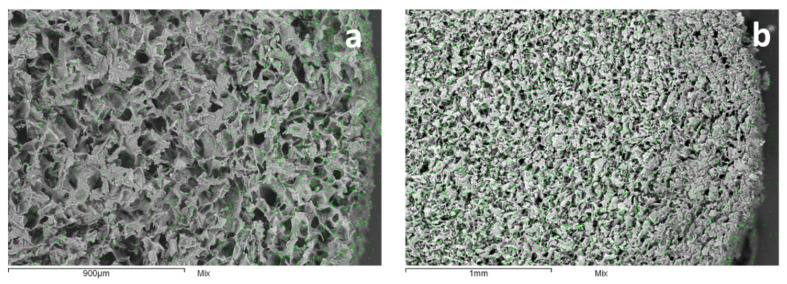
SEM–EDX (scanning electron microscopy–electron probe X-ray) image of a CNS cross-section after Zn(II) adsorption from 50 mg L^−1^ ASW (left, **a**) and 450 mg L^−1^ (right, **b**). Green dots represent metal ions.

No significant differences in pH were observed in ASW before and after CNS treatment during 24 h of exposure, and this was similar to the control and CNS only groups (*p* < 0.05) (Table S1). The SEM–EDX analysis evidences the limited diffusion kinetics of the ions in the intact sponge when operating at low metal salt concentrations (Figure 3a), while at higher concentrations, it is possible to verify the complete penetrability of the material, with active sites homogeneously distributed throughout the whole sponge (Figure 3b). CNS grinding allowed us to overcome this operative limit. The resulting powder consisted of particles with sizes in a range between 50 and 400 µm, with a maximum distribution at 130 µm.

The porosity of the CNS was determined in a previous work [38] by microcomputed tomography quantitative analysis and found to be about 70% of the bulk material. Moreover, in a more recent study [37], it was possible also to reveal the presence of nano-pores, thanks to a small angle neutron scattering (SANS) analysis, by investigating the water nanoconfinement geometries in the adsorbent material. The analysis of the experimental data allowed us to measure the short-range correlation length, which was measured to be in a range between 25 and 35 Å. We interpreted this data as the very first indirect evidence of the effective nano-dimension of the cavities, produced by the cross-linking of the reticulated cellulose nanofibers.

### 3.2. Effect-Based Study

#### 3.2.1. Cellular Bioassays

In the preliminary study, a dose-dependent increase in lysosomal membrane destabilization was observed in the hemocytes of mussels (16%, 41%, 100%) upon ZnCl_2_ exposure (1, 10 and 100 mg L^−1^). This allowed us to set the sub-lethal Zn(II) concentration at 10 mg L^−1^ as changes in NRRT resulted in levels significantly below the 50% of exposed cells without generating cell death [58,73] (Figure S2).

**Figure 4:**
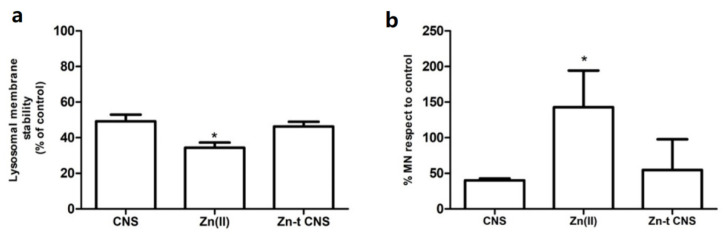
(**a**) Lysosomal membrane stability (LMS) shown as % vs. control (ASW) in mussel hemocytes at 30 min and (**b**) micronucleus frequency (MN) shown as % vs. control (ASW), after 48 h of exposure in the experimental groups: Zn(II) (10 mg L^−1^ of ZnCl_2_ in ASW); Zn-t CNS (ZnCl_2_ 10 mg L^−1^ contaminated ASW treated with CNS); CNS (ASW treated with only CNS). Results are shown as % towards controls (mussels exposed to ASW only). (*) indicates significant difference with respect to the Zn(II) group (*p* < 0.01).

In the CNS remediation study, a significant decrease in lysosomal membrane stability (LMS) (Figure 4a) was observed in the hemocytes of mussels exposed to ZnCl_2_ (10 mg L^−1^) compared to controls (*p* < 0.0001), while specimens exposed after CNS treatment showed a LMS similar to the controls (ASW) and to those exposed to CNS alone (*p* < 0.05).

Zn(II) at concentrations in the range of mg L^−1^ are known to cause severe damage in lysosomal membranes and therefore their stability has been widely used as a biomarker of exposure in field monitoring studies [61,79]. Dissolved Zn(II) is easily taken up through mussels’ gills and diffused across membranes and mantle syphons, while particulate forms are generally mistaken as food and accumulate in the digestive gland [48,55]. Consequently, Zn ions’ sequestration and toxic actions are closely connected to lysosomal function in bivalves, both in Zn accumulating organs as the digestive system and in single circulating immune cells (hemocytes) [47]. The recovery of lysosomal membranes’ stability to the levels of the controls in the mussels’ hemocytes after CNS treatment does confirm that the levels of Zn(II) in ASW were lowered until they were not able to destabilize the lysosomal membranes. In fact, the Zn level measured in exposure waters after CNS treatment after 24 h was 0.510 ± 0.0258 mg L^−1^, for which no significant effect on LMS was previously observed in mussels in our preliminary study (Figure S1). Furthermore, although CNS treatment did not reduce Zn levels to those of ASW (0.005 ± 0.001 mg L^−1^), such a concentration (0.510 ± 0.0258 mg L^−1^) was proven not to be effective in causing lysosomal membrane destabilization in the hemocytes of exposed mussels. Background levels of Zn in marine coastal waters are commonly below 1 mg L^−1^, for which no toxicity for marine bivalve species has been reported [15,80].

**Figure 5:**
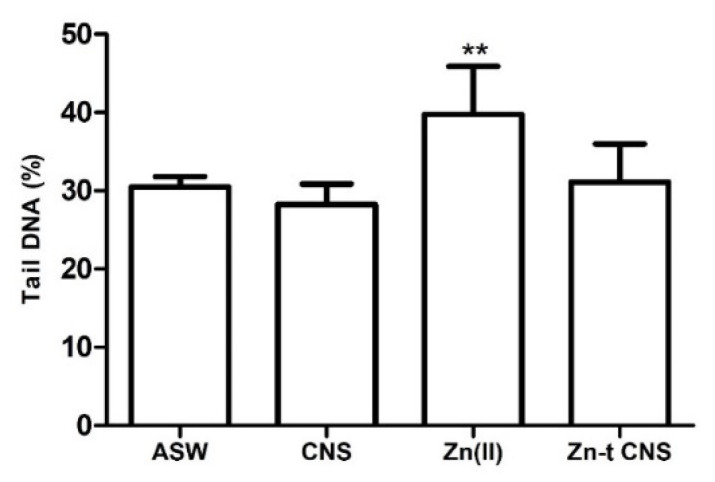
DNA primary damage (% tail DNA) in mussels’ gill cells after 48 h of exposure to the following experimental groups: Zn(II) (10 mg L^−1^ of ZnCl_2_ in ASW); Zn-t CNS (ZnCl_2_ 10 mg L^−1^ contaminated ASW treated with CNS); CNS (ASW treated with only CNS). Results are shown as mean ± SD. (**) indicates significant differences with respect to the control group (ASW) (*p* < 0.001).

**Figure 6:**
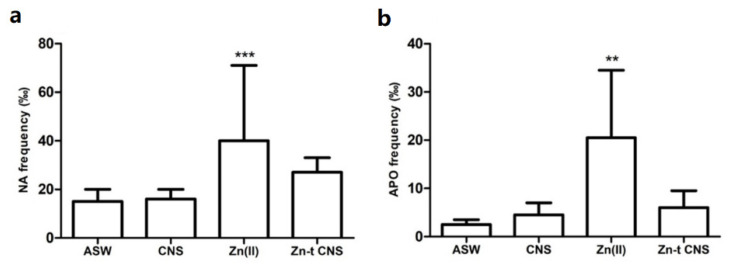
Frequencies (‰) of nuclear abnormalities (NA) (**a**) and apoptotic cells (APO) (**b**) in mussels’ gills after 48 h of exposure to the following experimental groups: ASW (control); Zn(II) (10 mg L^−1^ of ZnCl_2_ in ASW); Zn-t CNS (ZnCl_2_ 10 mg L^−1^ contaminated ASW treated with CNS); CNS (ASW treated with only CNS). Results are shown as mean ± SD. (**) indicates significant differences with respect to the control group (ASW) (*p* < 0.001). (***) indicates significant differences with respect to the control group (ASW) (*p* < 0.0001).

As observed for lysosomal membranes, a significant decrease in MN frequency (*p* < 0.001) was found in the hemocytes of mussels exposed after CNS treatment compared to those exposed to ZnCl_2_ (*p* < 0.0001); MN frequencies were also similar to those of the controls (ASW) and of specimens exposed to CNS only (Figure 4b). The same was observed in hemocytes; DNA strand breaks were significantly lower in the gills of mussels exposed after CNS treatment when compared to those exposed to ZnCl_2_ (*p* < 0.05), and DNA strand breaks were comparable in mussels exposed after CNS treatment, both in controls and in those exposed to CNS only (Figure 5). Approximately 3.5-fold more nuclear abnormalities (NA) were recorded in the gill cells of mussels exposed to ZnCl_2_ (*p* < 0.001), compared to specimens exposed after CNS treatment, controls and CNS-exposed only (Figure 6a). In addition, a ten-fold higher frequency of apoptotic cells (APO) was found in ZnCl_2_-exposed mussels’ gill cells compared to the controls, and no further differences were observed among mussels exposed after CNS treatment and those exposed to CNS only (Figure 6b).

ZnCl_2_’s ability to induce DNA strand breaks has been documented in the circulating and tissue cells of *M. galloprovincialis* [56,57]. However, upon CNS treatment, the DNA integrity of mussels’ gill cells was comparable to that of the controls and the mussels exposed to CNS alone.

As further confirmation, at the chromosomal level, the full recovery of DNA damage was observed in mussels exposed after CNS treatment. Heavy metals are known genotoxicants for marine bivalves, and NA in gill cells have been widely recognized as a suitable marker of DNA damage in *M. galloprovincialis* [63,81].

Genotoxicity can inhibit the cell cycle which influences the formation of MN [82]. The clastogenic activity of ZnCl_2_, by increasing the MN frequencies in the gill cells of mussels after acute short-term exposure conditions (48 h) and lower concentrations (0.17 mg L^−1^), has been reported [83]. The observed significant decrease in hemocyte MN and NA frequencies in mussels’ gill cells upon CNS treatment confirms the efficient removal of Zn(II) from ASW to levels which are not able to cause genotoxicity to mussels.

#### 3.2.2. Mantle Histology and Histochemistry

After 24 h, ZnCl_2_-exposure waters were slightly pink in color and the presence of suspended small tissue debris was confirmed by filtrates recovered with cellulose filters (0.45 µm), as shown in Figure S3. Exposure waters after CNS treatment, as well as controls (ASW) and those treated with only CNS, were transparent and no tissue debris was found upon filtration with cellulose filters (Figure S3).

**Figure 7:**
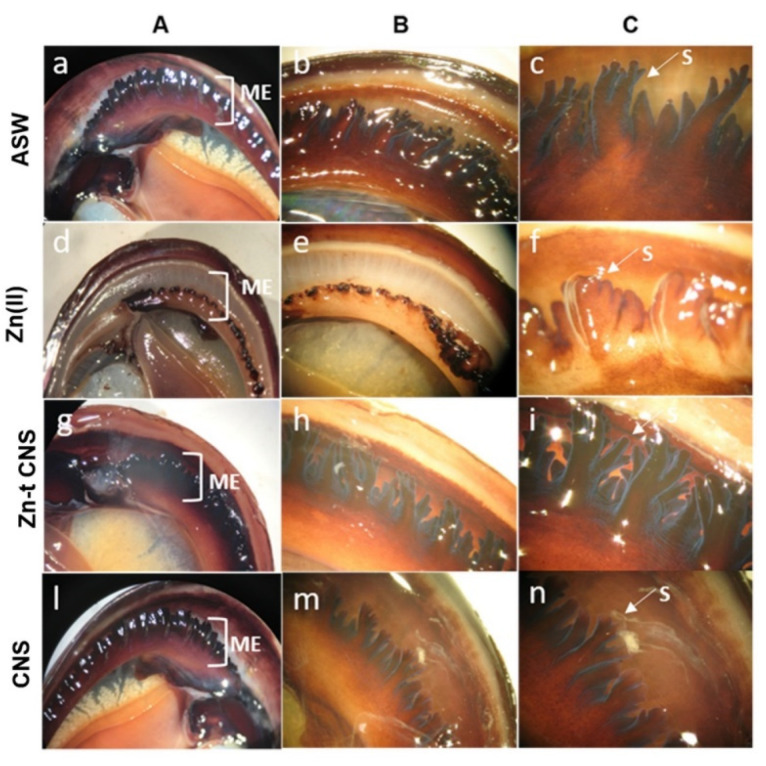
Mussels’ mantle, mantle edges (ME) and syphons (S) of specimens exposed to the following experimental groups: ASW (**a**–**c**, controls); Zn(II) (**d**–**f**, 10 mg L^−1^ of ZnCl_2_ in ASW); Zn-t CNS (**g**–**i**, ZnCl_2_ 10 mg L^−1^ contaminated ASW treated with CNS); CNS (**l**–**n**, ASW treated with only CNS). Images obtained with a Zeiss Stemi SV6 (8–50× magnification) binocular stereo microscope, magnification A (8×) B (10×) C (20×).

Mussels exposed to ZnCl_2_ (10 mg L^−1^) showed severe damage on mantle edges, with syphons reduced in length and/or wilted and fused together (Figure 7d–f). On the contrary, mussels exposed after CNS treatment (Figure 7g–i) showed an intact mantle margin and syphons were distended and vigorous. A similar mantle morphology was present in the control mussels (Figure 7l–n) and in those exposed to CNS alone (Figure 7a–c), suggesting the absence of any of those damages caused by ZnCl_2_ exposure.

External physical-chemical barriers, like shell, mantle and mucus, provide an important first line of defense in mollusks upon toxic chemical exposure. Zn(II) levels have been reported to exceed in the mantle edge compared to the rest of the mantle tissue, probably due to it being more directly in contact with seawater [43]. The lysosomes of mantle epithelial cells have been recorded to accumulate heavy metals [49,84], which can be stored in interstitial storage tissues and hemocytes [44,49,85]. Consequently, the mantle margin is more sensitive to Zn(II) exposure and, although acting as a physical barrier, it could be more damaged upon waterborne exposure than other internal organs such as the digestive gland [60]. By damaging syphons, which represent the mantle’s functional structures, as observed in ZnCl_2_-exposed mussels (ruptured, wilted or fused together), their protective role towards contaminant uptake can be seriously compromised [86]. The mantle of *Mytilus* also plays a major role in shell formation and the margin is considered the most active zone for shell deposition [87]. Cilia destruction in the outer epithelial cells of the mantle margin, as observed in mussels upon Zn-exposure, could affect the dynamic activity and movement of the organ itself [60].

**Figure 8:**
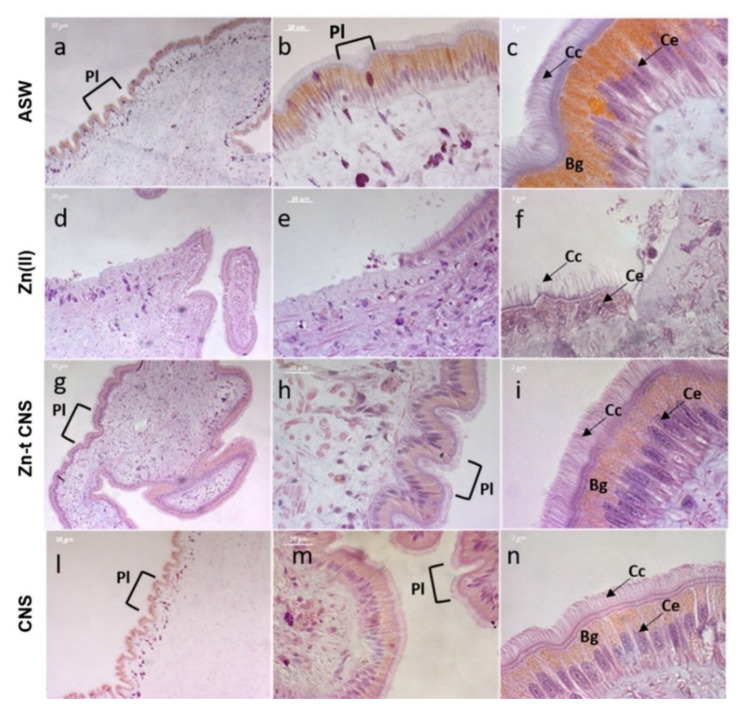
Light microscopy of mussels’ mantle edges stained with haematoxylin and eosin (H&E) of specimens exposed to the following experimental groups: ASW (**a**–**c**, control); Zn(II) (**d**–**f**, 10 mg L^−1^ of ZnCl_2_ in ASW); Zn-t CNS (**g**–**i**, ZnCl_2_ 10 mg L^−1^ contaminated ASW treated with CNS); CNS (**l**–**n**, ASW treated with only CNS). **Cc**: Ciliated columnar cells; **Ce**: columnar epithelium; **Bg**: brown intracellular granules; **Pl**: plicae. Images obtained with a Zeiss Axiophot epifluorescent microscope with AxioCamMRc5.

The H&E staining method further confirmed the severe damage on the mantle edges caused by ZnCl_2_ exposure. The columnar epithelium was fragmented along the entire margin and several holes were present (Figure 8d,e). In addition, epithelial cells were shortened, and the microvilli were fewer in number and were sagging and sparse (Figure 8f). Conversely, an intact columnar epithelium with elongated cells and with plicae along the edge was present in mussels exposed after CNS treatment (Figure 8g–i). A similar intact morphology was observed in the controls (Figure 8a–c) and in mussels exposed to CNS only, thus confirming a significant reduction in Zn damage on mantle edges in mussels exposed after CNS treatment (Figure 8l–n). In addition, columnar epithelial cells were well characterized by a monochromatic nucleus located at the basal area and several brown intracellular granules. Microvilli were homogeneously distributed, extended and elongated (Figure 8c,i,n).

**Figure 9:**
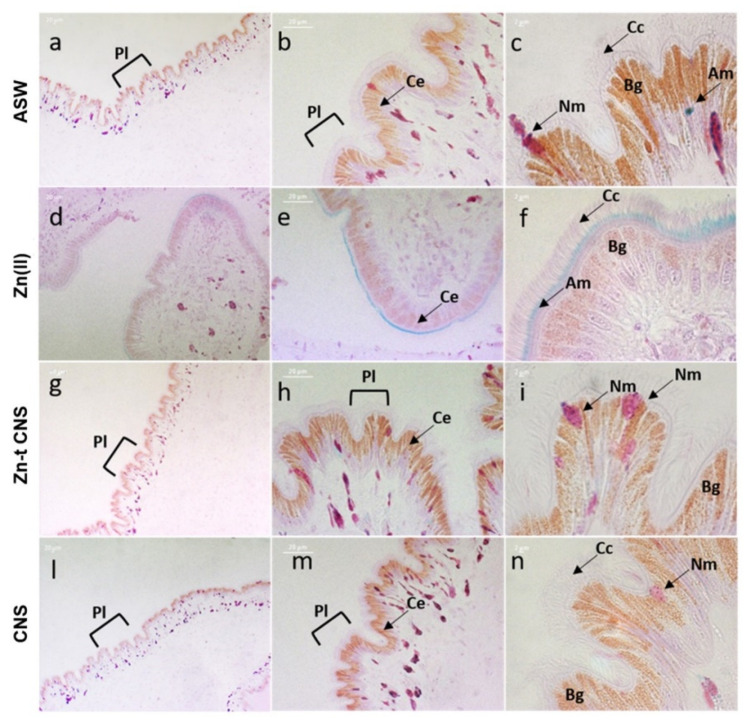
PAS–AB (periodic acid Schiff–Alcian blue) staining of mussels’ mantle edge cells of specimens exposed to the following experimental groups: ASW (**a**–**c**, control); Zn(II) (**d**–**f**, 10 mg L^−1^ of ZnCl_2_ in ASW); Zn-t CNS (**g**–**i**, ZnCl_2_ 10 mg L^−1^ contaminated ASW treated with CNS); CNS (**l**–**n**, ASW treated with only CNS). **Cc**: Ciliated columnar cells; **Ce**: columnar epithelium; **Bg**: brown intracellular granules; **Nm**: neutral mucosubstances; **Am**: acidic mucosubstances; **Pl**: plicae. Images obtained with a Zeiss Axiophot epifluorescent microscope with AxioCamMRc5.

All mussels, except those exposed to ASW only (controls), secreted a copious amount of mucus that settled to the bottom of the exposure tank. After 48 h of exposure, mussels’ valves were slimy and sticky, particularly in those exposed to ZnCl_2_ (data not shown). The characterization of mucus by PAS–AB highlighted the presence of two types of mucopolysaccharides in mussels’ mantle edges (Figure 9). Neutral mucopolysaccharides (purple color) are PAS positive and AB negative, and they present low viscosity, while acid mucopolysaccharides (blue color) are PAS negative and AB positive, and they present high viscosity [88]. In ZnCl_2_-exposed mussels, acidic mucosubstances were more present along the entire mantle margin, showing strong blue staining intensity, while the neutral ones were almost absent (purple staining) (Figure 9d–f). On the contrary, mussels exposed after CNS treatment showed an equal ratio (1:1) of acidic mucopolysaccharides and neutral ones (Figure 9g–i) and, furthermore, their distribution within the connective tissue was similar to that of the controls (Figure 9a–c) and of those exposed to CNS alone (Figure 9l–n).

The continuous and unrestricted release of mucus secretion is considered a typical generalized stress response in bivalves that helps them to regulate metal levels in the tissues by acting as a purifier [11,89]. In bivalves, Zn(II) exposure has been described as inducing an overproduction of mucus, which is considered an inflammatory reaction that limits metal absorption or increases its excretion [43,52,90]. Mucus is a slimy and viscous secretory product that is involved in several physiological and behavioral functions, like the maintenance of internal water homeostasis, nutrition, immune defense and lubrification-related activities [86]. A role in isolating mussels from their environment and better counteracting external stressors has been also hypothesized [91]. Excessive mucus excretion may often lead to tissue desiccation and immune dysfunction due to the reduction of the internal mucosal sheath, which acts as a barrier to pathogens and toxins [86,92,93]. The higher secretion of acidic mucosubstances than of neutral ones in ZnCl_2_-exposed mussels has already been described as a mussel’s response to metal toxicity [94,95]. In addition, an increase in mucus-secreting cells and non-ciliated epithelial cells in the sub-epithelial region of the mantle margin, such as that observed in Zn-exposed mussels, have been linked to exposure to chemical stressors [48,60,89]. Therefore, the excess mucus production in mussels exposed after CNS treatment revealed their responsiveness to low levels of Zn, such as those still present in ASW after CNS treatment (0.510 ± 0.025 mg L^−1^). Such evidence, together with other toxicological data obtained on mussels exposed after CNS treatment, supports the suitability of the proposed biological effect-based approach to assess the efficacy of CNS in reducing Zn toxicity in mussels.

Only very recently, aquatic ecotoxicology has begun to move toward the environmental safety of (nano)materials, including those for pollution remediation (nanoremediation), and related challenges in order to provide suitable testing strategies and methods to prevent side effects [21,96,97,98,99,100,101,102,103,104]. Such scientific gaps call for a thorough evaluation of their environmentally safe application and, therefore, a case-by-case analysis must be undertaken to assess their actual applicability [105,106]. While in our previous study [40], we aimed to develop an eco-design strategy to improve CNS’ ecosafety by modifying both the formulation and purification protocols, here, we used a more complex effect-based approach to assess their efficacy in Zn(II) removal from seawater to levels which do not cause any harm to marine species [107,108]. The revealed sensitivity of the effect-based approach thus represents a promising solution to overcome the current limitations of nanotechnologies for environmental remediation and the reasonable policy concerns due to its in-situ application [19,21,22,23]. However, this clearly represents a first attempt to promote the applicability of an effect-based approach as ecotoxicological proof of the material’s efficacy, which is commonly based on analytical chemistry validation. To this aim, in our study, the almost complete removal of Zn(II) upon CNS treatment and, respectively, of 91.7% and 91.5% at time zero and after 24 h, was confirmed by analyzing Zn levels in exposure waters (Table 1). A significant reduction in the Zn(II) concentration was generally observed in all experimental groups after 24 h, including ASW, except for waters treated with CNS, which showed a slight increase (Table 1).

The uptake of Zn(II) by mussels during the 24 h of exposure due to seawater filtration might have also reduced their levels in ASW. Adult mussels can easily filter up to 3 L in at least 1 h and reach a quick state-state in heavy metal accumulation in their soft tissues in proportion to their concentration in seawater [46,109]. This peculiar behavior of filter-feeders towards heavy metals in the water column demonstrates the need to further implement the effect-based approach by testing other species from different trophic levels (e.g., bottom-dwellers, benthic grazers), as well as the need to include more sensitive and/or target ones of specific pollutants to be remediated. Long-term exposure scenarios should also be included, which will allow us to reach a more comprehensive view of the potential ecological disturbances and assessment of the adverse effects on marine ecosystems. Field-scale application, for instance, by using natural seawater media and mesocosm settings, will also elucidate potential confounding factors which could affect the material’s behavior and efficacy (i.e., dissolved organic carbon and colloids on adsorption ability), thus improving the environmental relevance of the assessment and linking ecotoxicological and chemical information.

## 4. Conclusions

Our findings demonstrate that after CNS treatment, Zn(II) toxicity was significantly reduced in marine mussels, both at cellular and tissue levels, which was in agreement with the significantly reduced Zn(II) nominal levels (>90%) in exposure waters. Upon CNS treatment, Zn-induced genotoxicity in circulating immune and gill cells was significantly reduced to that which was observed in specimens exposed to ASW (controls), as well as histological and cytological damage in mantle edge and epithelial cells. The proposed effect-based approach was thus proven to be useful in further supporting the environmental relevance of CNS’ efficacy in heavy metal removal from seawater and their ecosafe application by linking ecotoxicological and chemical information.

## Figures and Tables

**Table 1:** Zn(II) concentration (mg L^−1^) in mussels’ exposure waters at time zero (*T*_0_) (soon after preparation) and after 24 h (*T*_24h_) of the following experimental groups: Zn(II) (ZnCl_2_ 10 mg L^−1^ in ASW); Zn-t CNS (ZnCl_2_ 10 mg L^−1^ contaminated ASW treated with CNS), CNS (ASW treated with CNS only); ASW (control). Results are reported as mean ± SD. (*) indicates significant differences with respect to Zn(II) and (**) with respect to controls (ASW) (*p* < 0.001).

Exposure Groups	Zn(II) *_T_*_0_	Zn(II) *_T_*_24h_
Zn(II)	8.567 ± 0.185 **	6.006 ± 0.604 **
Zn-t CNS	0.710 ± 0.0568 *	0.510 ± 0.0258 *
CNS	0.00251 ± 0.0001 *	0.0245 ± 0.0008 *
ASW	0.0047 ± 0.0006	0.0005 ± 0.0001

## Supplementary Materials

The following are available online at https://www.mdpi.com/2079-4991/10/7/1283/s1. Table S1: pH levels in mussel’s exposure media. Figure S1: Lysosomal membrane destabilization in mussels exposed to ZnCl_2_; Figure S2: Nuclear abnormalities detected in mussel’s gill cells; Figure S3: water exposure media filtered using 0.45 µm cellulose filter paper.
