# Supplementary material for: Effect-Based Approach to Assess Nanostructured Cellulose Sponge Removal Efficacy of Zinc Ions from Seawater to Prevent Ecological Risks
Source: Nanomaterials (Basel). 2020 Jun 30;10(7):1283. doi: 10.3390/nano10071283 (PMC7407410; doi:10.3390/nano10071283)
Supplement: Supplementary file 1 [file nanomaterials-10-01283-s001.pdf]

Supplementary material

Effect-based approach to assess nanostructured cellulose sponge removal efficacy of Zinc ions from seawater to prevent ecological risks

Giulia Liberatori [1], Giacomo Grassi [1], Patrizia Guidi [2], Margherita Bernardeschi [2], Andrea Fiorati [3], Vittoria Scarcelli [2], Massimo Genovese [2], Claudia Faleri [4], Giuseppe Protano [1], Giada Frenzilli [2,*], Carlo Punta [3] and Ilaria Corsi [1,*]

[1] Department of Physical, Earth and Environmental Sciences and INSTM Local Unit, University of Siena, 53100 Siena, Italy; giulia.liberatori@student.unisi.it (G.L.); grassi23@student.unisi.it (G.G.); giuseppe.protano@unisi.it (G.P.)

[2] Department of Clinical and Experimental Medicine-section of Applied Biology and Genetics, University of Pisa, 56126 Pisa, Italy; patrizia.guidi@unipi.it (P.G.); margherita.bernardeschi@unipi.it (M.B.); vittoria.scarcelli@unipi.it (V.S.); massimo.genovese@unipi.it (M.G.)

[3] Department of Chemistry, Materials, and Chemical Engineering "G. Natta" and INSTM Local Unit, Politecnico di Milano, 20131 Milano, Italy; andrea.fiorati@polimi.it (A.F.); carlo.punta@polimi.it (C.P.)

[4] Department of Life Sciences, University of Siena, 53100 Siena, Italy; faleric@unisi.it

* Correspondence: ilaria.corsi@unisi.it (I.C.); giada@biomed.unipi.it (G.F.); Tel.: +39-0577-232168 (I.C.); +30-050-2219111 (G.F.)

Number of figures: 3

Number of tables: 1

Number of pages: 4

Tab. S1. pH levels measured in the water media of both experiments at T_0 and T_{24h} in the groups: ASW (control); Zn(II) 1, 10, 100 mg L^{-1} in ASW; CNS (ASW treated with only CNS); Zn(II)(10mg L^{-1} in ASW); Zn t-CNS (Zn(II) 10mg L^{-1} after CNS treatment).

	**Experimental groups**	**T_0**	**T_{24h}**
**$ZnCl_2$ sub-lethal effect conc. Exposure study**	ASW	7.94 ± 0.07	7.96 ± 0.16
	Zn(II) 1 mg L^{-1}	7.89 ± 0.07	7.9 ± 0.12
	Zn(II) 10 mg L^{-1}	7.78 ± 0.07	7.83 ± 0.17
	Zn(II) 100 mg L^{-1}	7.45 ± 0.08	7.48 ± 0.06
**Effect-based study on CNS adsorption ability**	ASW	7.70 ± 0.14	7.72 ± 0.18
	CNS	8.32 ± 0.26	8.04 ± 0.35
	Zn(II)	7.53 ± 0.14	7.70 ± 0.10
	Zn t-CNS	8.12 ± 0.28	8.01 ± 0.13

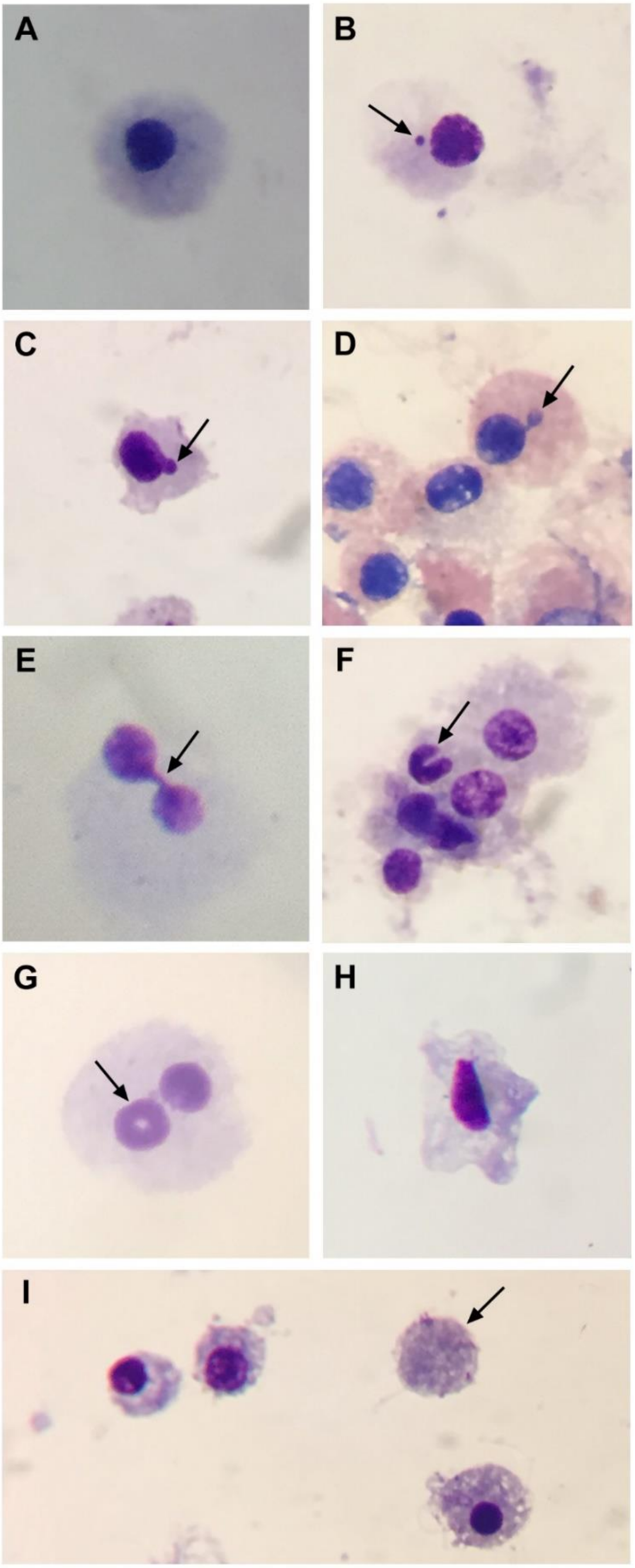

Fig. S1. Nuclear abnormalities (NA) observed in mussel gill cells (stained with 6% Giemsa). (A) Control cell. (B) Cell with micronucleus. (C) Bleb. (D) Bud. (E) Nuclear bridge. (F) Notched nucleus. (G) Circular nucleus. (H) Lobed nucleus. (I) Anisochromatic cell.

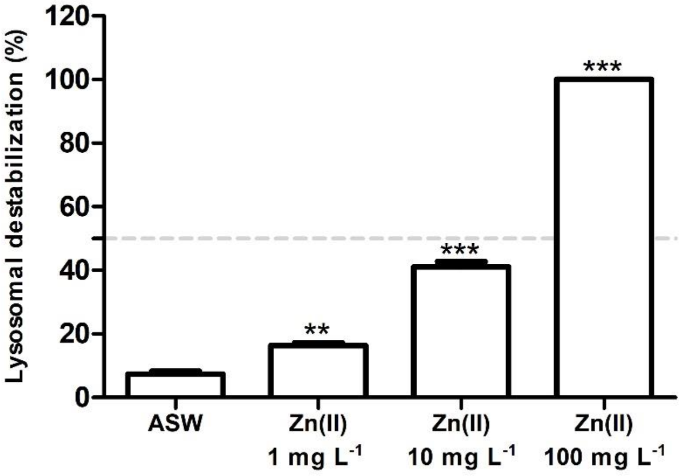

Fig. S2. Percentage of lysosomal membranes destabilization in mussel hemocytes after 48h of exposure in the following experimental groups exposed to $ZnCl_2$ (1, 10, 100 mgL^{-1} in ASW). The dashed line indicates the reading limit of the destabilized cells (50%). Results are reported as mean ± SD. (***), (**) indicates significant differences respect to the control group, corresponding to $p < 0.0001$ and $p < 0.001$ respectively.

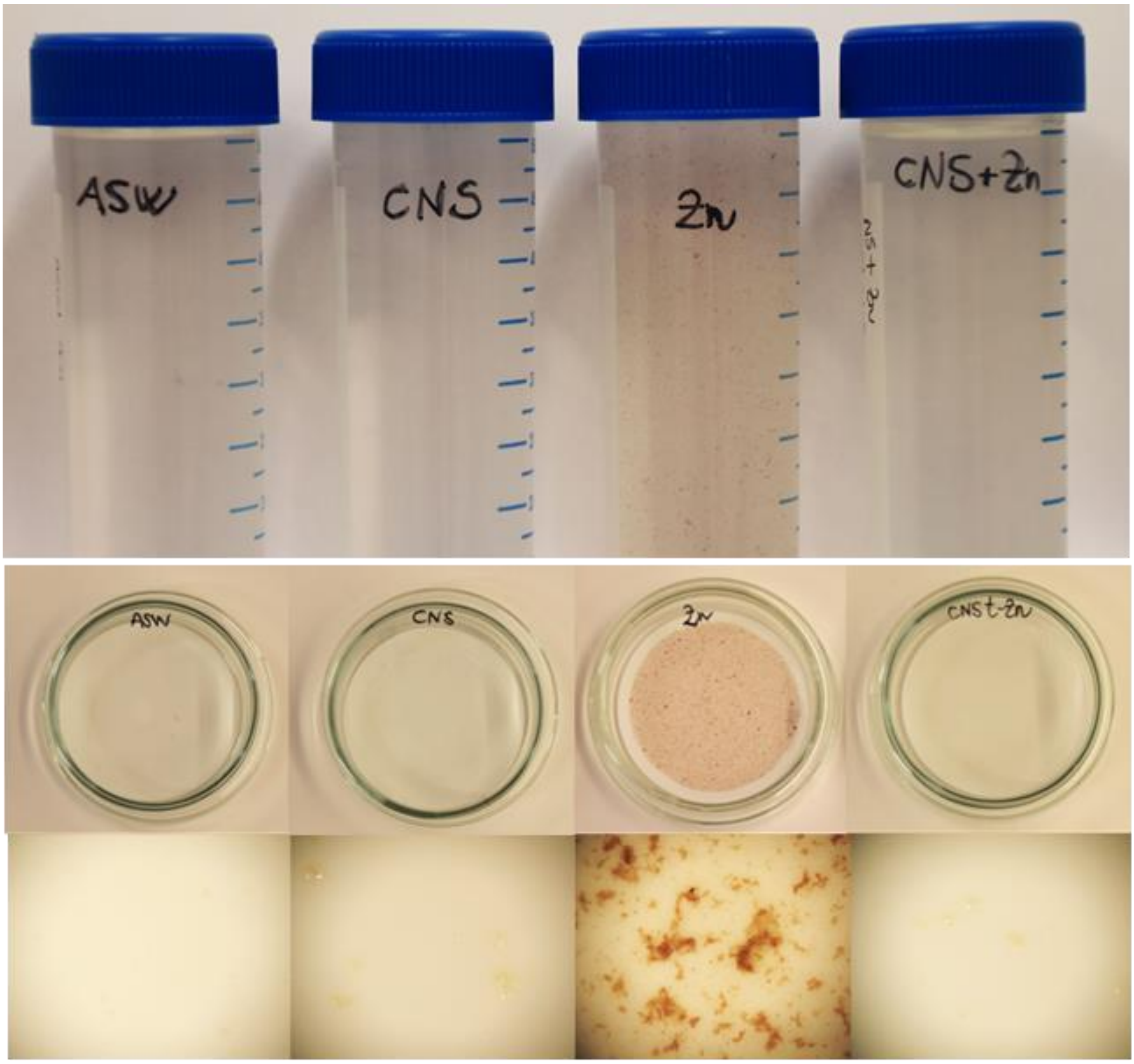

Fig. S3. Falcon tubes containing water exposure media after 24h (T_{24h}) of the following experimental groups: ASW (control); CNS (ASW treated with only CNS); Zn(II)($ZnCl_2$ 10mg L^{-1} contaminated ASW); CNS t-Zn ($ZnCl_2$ (10mg L^{-1}) contaminated ASW after CNS treatment). Details on 0.45 µm filter cellulose paper and at higher magnification (40×) under light microscope.
